# A study of error diversity in robotic swarms for task partitioning in foraging tasks

**DOI:** 10.3389/frobt.2022.904341

**Published:** 2023-01-04

**Authors:** Edgar Buchanan, Kieran Alden, Andrew Pomfret, Jon Timmis, Andy M. Tyrrell

**Affiliations:** ^1^ School of Physics, Engineering and Technology, University of York, York, United Kingdom; ^2^ School of Computer Science, University of Sunderland, Sunderland, United Kingdom

**Keywords:** swarm robotics, fault tolerance, error diversity, task partitioning, foraging

## Abstract

Often in swarm robotics, an assumption is made that all robots in the swarm behave the same and will have a similar (if not the same) error model. However, in reality, this is not the case, and this lack of uniformity in the error model, and other operations, can lead to various emergent behaviors. This paper considers the impact of the error model and compares robots in a swarm that operate using the same error model (uniform error) against each robot in the swarm having a different error model (thus introducing error diversity). Experiments are presented in the context of a foraging task. Simulation and physical experimental results show the importance of the error model and diversity in achieving the expected swarm behavior.

## 1 Introduction

Robot swarms are capable of performing different tasks in an efficient and decentralized way, in part, due to their high level of parallelization. In the past, swarm robotic systems were assumed to be inherently robust to failures due to their high degree of robot redundancy. However, Winfield and Nembrini (2006) demonstrated that this is not always the case and that specific processes must be introduced to increase the robustness of the swarm. Bjerknes and Winfield (2012) demonstrated that in various scenarios, specific partial failures in robots can lead to task degradation or, in the worst case, the task not being achieved.

Fundamental mechanisms to achieve robust fault-tolerant swarm robotic systems have been considered by different authors including detecting faults (Christensen et al., 2009; Tarapore et al., 2015), diagnosing the fault type (Li and Parker, 2007; Keeffe et al., 2017), recovering from the fault (Humza et al., 2009; Timmis et al., 2010), or a combination of all of these fault-tolerant methods (Parker, 1998; Parker and Kannan, 2006). However, in all of these works, the authors classify the robots according to their behavior, that is, a robot being either faulty or non-faulty, where faulty robots exhibit abnormal behavior compared with non-faulty robots. However, in reality, it is often hard to distinguish between a faulty and non-faulty robot due to the diversity of errors across the swarm.

The importance of considering error diversity in swarm robotics can be summarized in three points. 1) It is possible to reduce the reality gap between swarm behaviors in simulation and swarm behaviors in hardware. 2) The error non-diversity could lead to false positive results and missing critical faults that ultimately led to failures. 3) It is important for each robot to learn and adapt to its own inherent error; in this way, the robot swarm will exhibit better performance.

To the best of the authors’ knowledge, there is no literature where diversity in terms of levels of error is considered in fault-tolerance experiments. Most related research examines diversity across the swarm from an evolutionary perspective, where controllers can be evolved independently for each robot in the swarm. For example, reinforcement learning has been used to train task-specialized robots where each robot in the swarm learns to perform a specific task (Balch, 1998; Li et al., 2003; Balch, 2005) or tasks have been previously allocated to robot members (Zhang et al., 2008). Robot controllers are generally evolved with two methods: genetically homogeneous or genetically heterogeneous (Bongard, 2000; Potter et al., 2001; Trianni and Nolfi, 2011; Tuci, 2014; Hart et al., 2018). In the homogeneous method, the controller is expressed as a single genotype, which is then cloned into each robot in the swarm. With respect to the heterogeneous method, each robot has its own genotype, and after evolution, each robot has a specific role in the task. Despite this work on controller heterogeneous swarm systems, there is no research that considers the appropriate level of error for each robot during the simulation and its impact on the performance of the swarm in both simulation and hardware. In this paper, we identify and study the discrepancy of results when degrees of diversity of error are considered, referred in this paper as *heterogeneous error*, or if all the robots share the same degree of error, referred to as *homogeneous error*.

The task considered in this paper is task partitioning in foraging. Task partitioning, first observed in biological systems (Ratnieks and Anderson, 1999), has been used in the swarm robotics context to prevent bottlenecks close to the home area where the items are deposited (Goldberg and Matarie, 2001; Brutschy et al., 2014; Pini et al., 2011). Other approaches such as Pini et al. (2013), Pini et al. (2014), and Buchanan et al. (2016) have focused on fault tolerance in order to increase performance when the dead-reckoning error is present in a robot. Task partitioning has also been used to study the effect of task decomposition on emergent swarm intelligence (Harwell et al., 2020).

Task partitioning is a technique that divides a single task into multiple smaller subtasks, with the objective of reducing the amount of distance traveled by each robot and, thus, the error in dead reckoning. A robot finds an item in the environment and transports the item toward the home area for a short distance referred to as the partition length (*P*). Then, the item is exchanged by either leaving the item on the floor for a different robot to collect (indirect transfer) or waiting for the second robot to receive the item directly from the first robot (direct transfer). Since the robots are traveling a shorter distance *P* compared to the total distance between the home area and the items’ source, the dead reckoning is smaller. The amount of distance *P* depends on the approach taken.

The experimental framework in this paper follows a top-down approach from the macro to the micro perspective, combined with three layers of abstraction: emulation, simulation, and hardware.

In the first stage, emulation, an ensemble of different machine learning techniques is trained with a dataset generated from Latin hypercube sampling in simulations. The main advantage of using emulation is to save time and computation resources, compromising the resolution of the simulation. In other words, the emulation is used to explore the experiments from a macro or global perspective, which is only concerned with the behavior of the swarm as a whole. Emulation is generated for each strategy with each error type.

The second and third stages, simulation and hardware, are studies from the micro or local perspective which is concerned with the contribution of each robot to the task. This aids in obtaining a comprehensive understanding of the behavior of the swarm.

The contributions are summarized as follows:

• Demonstration that the assumption made that all the robots in a simulated robotic swarm shared the same error model can lead to unexpected swarm behaviors when testing the behavior with physical robots where each robot experiences a different error
• Difference in behaviors introduced by error diversity can be mitigated by having each robot learn the task according to the success of its performance, thus reducing errors for all robots

The hypotheses explored in this paper are as follows:

1. The consideration of error diversity leads to different correlation values than non-diversity
2. Each robot adapts differently to its inherent error

The rest of the paper is organized as follows. In Section 2, a case study is presented and each task partitioning strategy is described. The methodology followed in the experiments for this study is described in Section 3. Experiments and comments on results are presented in Section 4, and Section 5 provides the summary and conclusion.

## 2 Case study: Foraging task partitioning strategies

Foraging with dead reckoning as navigation is used as a task to study the impact of the error diversity and uniformity across the robots in the swarm to the behavior and performance of the swarm.

Within a simple foraging task, a group of robots explore the environment searching for items to collect. Then, after an item is found, the position is recorded by the robot, and finally, the robot transports the item toward the home area. This study focused on a single home area; however, this work could be used for multiple places at home (Lu et al., 2018).

Due to systematic and unsystematic errors in dead-reckoning navigation, the real-time position is often inaccurate leading to an error in the positioning estimation. Errors accumulate as a robot travels, which in turn affects the estimated item position. As a consequence, when the robot attempts to go back to where it found the last item in order to collect further items, it reaches a different location. One simple way to mitigate this error is to partition the distance traveled by each individual robot.

In this study, two task partitioning foraging strategies are used to examine the effects of error diversity and uniformity. The objective of this approach is to compare traditional foraging with a strategy where robots learn to divide the task into multiple smaller tasks, depending on the success, or otherwise, of item collection. Different emergent behaviors are expected to appear when error diversity and uniformity are considered for each strategy. In the first part of this section, the foraging task is described, and the first strategy, the non-partitioning strategy (NPS) (Pini et al., 2011), is introduced. A second strategy is then described as the dynamic partitioning strategy (DPS) (Buchanan et al., 2016), where the number of partitions is defined by a penalty and reward mechanism.

### 2.1 Foraging task and non-partitioning strategy

**FIGURE 1:**
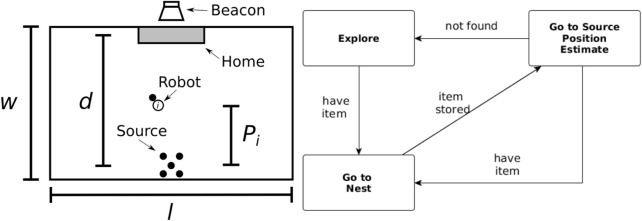
Foraging arena (left) and the finite state machine used by the robots (right). Left: the arena has a rectangular shape defined by its width (w) and length (L). The distance between the home area and the items’ source is represented by *d*. *P*_*i*_ represents the individual partition length for robot *i*. Right: non-partitioning strategy finite state machine composed of three states: *explore*, *go-to-nest*, and *go-to-source*.

In both strategies, contained in the environment is a virtual beacon that guides the robots toward their home area (nest). The arena, shown in Figure 1, is rectangular and is defined by its width (w) and length (L) and the distance between the home area and the items to be collected [source (*d*)].

In the non-partitioning strategy (NPS), the robots take the items directly from the item source to the home area. The controller is a finite-state machine (FSM) composed of three states as shown in Figure 1, and each state is described as follows.

First, the robot starts in the *explore* state in which all the robots are searching for items in the arena. The exploration consists of a motor schema-based navigation (Arkin, 1987) with two behaviors: 1) stay on the path and 2) avoid obstacles. Once an item is within the range of vision of the robot, it records the position, picks up the item, and then, enters the *go-to-nest* state.

In the *go-to-nest* state, the robot travels toward the home area by following the virtual beacon. The robot aligns itself toward the source of the light and moves straight to that location. The robot knows it has reached the home area when ground sensors on the robot detect a change in color. The item is deposited, and the robot transitions to the *go-to-source* state.

The *go-to-source* state consists of the robot traveling back to where the estimated position of the items’ source is recorded. The robot uses dead reckoning as navigation to guide itself to reach the item’s source. If an item is found, the robot picks up another item and switches to *go-to-nest* state; if the robot does not find an item, then it changes to the *explore* state.

In an ideal scenario, the robot would be continuously retrieving items from the source without transitioning to the *explore* state. However, in reality, robots are susceptible to systematic and non-systematic errors introduced by the dead-reckoning noise, which in turn affect target position estimation. The error accumulates the longer the distance a robot travels; therefore, the probability of finding the items source decreases as *d* increases due to the drift from the actual items’ source and the estimated item’s source position.

In order to decrease the error introduced by dead-reckoning noise, the task can be decomposed into smaller subtasks performed by each robot, and this is described in the following section.

### 2.2 Dynamic partitioning strategy

In dynamic partitioning strategy (DPS), each robot *i* changes its individual distance traveled (*P*_*i*_) using a penalty and reward mechanism. Every time a robot finds an item in the *go-to-source* state, *P*_*i*_ is increased. If the robot *i* does not find an item, then *P*_*i*_ is decreased.

*P*_*i*_ is calculated with Eq. 1 where *k* defines the amount of distance changed to *P*_*i*_ and *α* defines the ratio between reward and penalty. As *α* decreases, the robot is rewarded. *P*_*i*_(*t*) is the partition length after the application of the penalty and rewards, and *P*_*i*_(*t*−1) is the value beforehand.

$$P_{i}\left(t\right)=\left\{\begin{array}{cc} P_{i}\left(t-1\right)+k\left(1-\alpha \right)\; & \;\text{if item found}\\ P_{i}\left(t-1\right)-k\alpha \; & \;\text{if item not found}. \end{array}\right.$$ (1)

## 3 Methodology

This section presents the methodology followed for all experimental work in the study. First, parameters and outputs are defined; then, the experimental framework is followed and the tools used are introduced; and finally, the statistical techniques used to analyze the experiments’ results are described.

### 3.1 Terms, parameters, and outputs

In order to understand the impact of having different error models for each robot in a swarm compared to all robots having the same error model, it is important to perform a study from both the macro (the swarm as a whole) and the micro (each robot by itself) perspectives.

From a macro perspective, the *total items collected* output provides a good performance metric for the swarm as a whole, and this can be compared with different *swarm sizes* and distances between the home area and the items’ source. The *social entropy* provides a metric for how homogeneous the swarm is, according to the different individual errors.

From a micro perspective, it is important that the outputs reflect the performance of each robot by itself. The *collection ratio* output represents how successful a robot is at finding the item, providing an indication of how bad the error is for each robot. In a similar way, the *explore ratio* provides an indication of the amount of time the robot spends exploring.

#### 3.1.1 Terms and parameters

To allow appropriate results to be collected and meaningful analysis to be undertaken, a number of environmental and system parameters must be defined for the experiments. The terms that are used for the experiments are *simulation length*, *swarm size*, and the parameters are *d*, *P*, and *α*.

##### 3.1.1.1 Terms

The *experiment length* term represents the duration of the experiment until it stops.

The *swarm size* term represents the total number of robots performing the task. The minimum *swarm size* is 2 because at least a pair of robots is required to have task partitioning. The maximum *swarm size* is 15, as with larger values the robots spend more time avoiding each other than collecting items for the given environments.

##### 3.1.1.2 Parameters

The distance between the home area and the items’ source is shown as *d*.

*Swarm density* is an implicit parameter explored in this study, which is correlated to the swarm size and *d*.

The *partition length (P)* parameter represents the distance that a robot travels from where it finds an item for the first time to its home area, measured in meters. All robots start with the same *P*.

The *α* parameter regulates the amount of penalty and reward assigned to each robot in the swarm for DPS.

#### 3.1.2 Outputs

The outputs collected and subsequently used to compare and contrast the various methods from the experiments are the *final* $\tilde{P}$ , *total items collected*, *explore ratio*, *collection ratio*, and *social entropy*. These outputs are described next.

The *total items collected* output represents the number of items collected at the end of the experiment.

*P*_*i*_, the *final* $\tilde{P}$ , represents the last median of $\tilde{P}$ of all from all the robots when the experiment stops from a uni-modal distribution.

The *explore ratio* represents the amount of time spent by the swarm in the *explore* state. The *explore ratio* is measured as $\frac{T_{E}}{T_{T}}$ where *T*_*E*_ represents the sum of the total time spent by all the robots undertaking iterations in the *explore* state and *T*_*T*_ is the *simulation length* multiplied by the *swarm size*.

The *collection ratio* isolates the frequency with which a robot successfully collects an item, allowing for the identification of a correlation between the level of error and how often a robot retrieves an item $\frac{I_{F}}{\left(I_{F}+I_{L}\right)}$ . *I*_*F*_ is a counter that records the number of times a robot transitions to the *go-to-nest* state from the *neighborhood exploration* state. *I*_*L*_ is a counter that records the number of times a robot enters the *explore* state from the beginning of the simulation.

*Social entropy* is a metric that measures diversity in robot swarms and was initially introduced by Balch (1998). This metric is used to measure robot homogeneity across the swarm according to the classification of robots by their individual performance, where a robot can classify as faulty (high error) or non-faulty (low error). Social entropy *H*(*R*) is calculated with Eq. 2, where *M* is the number of subsets (faulty and non-faulty robots), *p*_*i*_ is the proportion of agents in each subset, and *i* and *R* represent the group of robots. The lower the value of *H*(*R*) the more homogeneous the swarm is. This parameter’s output is explored in more detail in Section 4.2.2.

$$H\left(R\right)=-\overset{M}{\underset{i=1}{\sum }}p_{i}\log_{2}\left(p_{i}\right).$$ (2)

### 3.2 Experimental framework and tools

**FIGURE 2:**
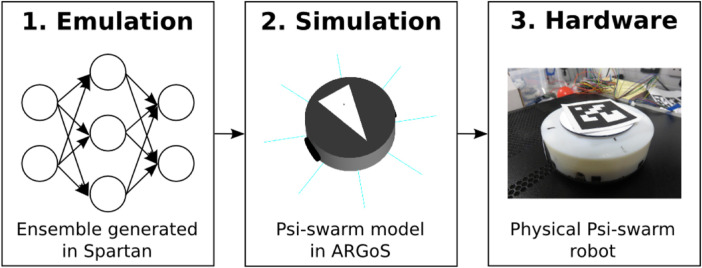
Framework for the experiments in this study. 1) Emulation is generated from different machine learning techniques in order to perform an enriched model analysis from a macro perspective which could not be possible to do in simulations due to constraints in time, battery span, and computational power. 2) Simulations are used to study the model from a micro perspective. 3) Experiments in hardware are used to validate the results shown in emulation and simulation.

The experimental framework is a top-down approach divided into three stages, as illustrated in Figure 2. The first stage consists of studying the effect of implementing heterogeneous and homogeneous errors from a macro perspective (considering the swarm as a whole) by performing a sensitivity analysis on each strategy *via* emulation. The second stage consists of studying the effects of heterogeneous and homogeneous errors from a local perspective (considering each robot as an individual) in simulation. Finally, experiments with physical robots validate the results from emulation and simulation.

#### 3.2.1 Simulation and hardware

The simulator used throughout the work described in this paper was ARGoS (Pinciroli et al., 2011), and this was selected due to the support provided to run experiments for large numbers of robots. For the experiments in this study, a simulated and real version of the psi-swarm robot platform (Hilder et al., 2014) (Figure 2) is used. This robot has infrared and ground color detector sensors. Since this robot does not have a camera, a virtual camera is used in hardware experiments instead to aid the robot in detecting the items. The virtual camera consists of a tracking system that retrieves the position of an ArUco tag (Garrido-Jurado et al., 2014) attached on top of each robot. A Bluetooth signal is sent to the robot to let it know that it has found an item.

The source code for the psi-swarm controller used in the experiments and detailed in this paper can be found online at https://github.com/edgarbuchanan/psiswarm_task_partitioning.

#### 3.2.2 Training data

A total of four datasets are generated from the outputs of the Latin hypercube sampling (Mckay et al., 1998) in simulations for each strategy (NPS and DPS) and for each error type (heterogeneous and homogeneous). On average, 5 h of simulated time represent roughly 1 min in real time. The number of replicates needed for the experiments shown in this paper is 180 (see Section 3.3 for more information). Therefore, for a set of experiments for a single strategy, it would take 3 h in real time. The amount of time required for experiments escalates if a population of samples and/or number of generations is required. Therefore, for parameter analysis that requires a large number of samples, the dataset can take a long time to produce.

#### 3.2.3 Emulator

Incorporating a combination of machine learning algorithms, an emulation is created that can be used as a surrogate for original simulations. This emulator is capable of making efficient predictions of simulation output for a given parameter set, reducing the time and resource requirements inherent in simulation due to the large number of replicates and size of the parameter space.

Parameters and outputs considered for the training of each emulator can be found in Table 1.

**TABLE 1:** Parameters and outputs used for the Latin hypercube sampling.

Parameters		
Strategy	Name	Interval
NPS	*Swarm size*	[2–14]
	*d*	[0.5–2.0 m]
DPS	*Swarm size*	[2–14]
	*d*	[0.5–2.0 m]
	*α*	[0–1]
Outputs		
	Name	Interval
	*Total items collected*	[0 max]
	*Explore ratio*	[0.1]
	*Collection ratio*	[0.1]

**FIGURE 3:**
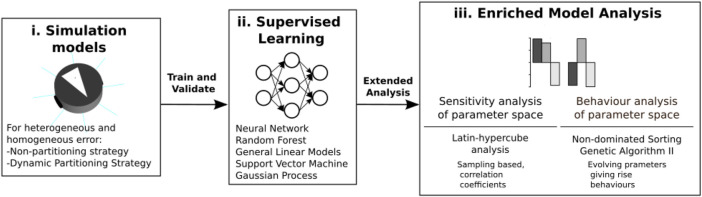
From simulation to emulation block diagram. A dataset is generated from Latin hypercube analysis in simulations (i). This dataset is used to train and validate the ensemble composed with different machine learning techniques (ii). Different statistical tools are used to analyze the ensemble model (iii).

The procedure to generate the emulator and use this to perform a predicted sensitivity analysis is as follows. For each parameter in each strategy, a value range is assigned and sampled using Latin hypercube sampling, which ensures adequate coverage of the parameter space (Figure 3i). Then, each of the four datasets is used to train and validate the performance of five machine learning techniques (neural network, random forest, general linear models, support vector machine, and Gaussian process).

These five individual emulators are combined to form one predictive emulation, or ensemble, where predictions are generated by weighing the performance of each algorithm on a test set (Figure 3ii). Combining the five algorithms has been shown to increase the accuracy of prediction over using each emulator in isolation (Alden et al., 2018).

Finally, the emulation is used to perform an enriched sensitivity analysis of the parameter space (Figure 3ii). Using sensitivity analyses, important parameters are revealed for each strategy, the understanding of the relationships between parameters and outputs with each is increased, and regions can be found for the parameters with maximum and minimum performance.

The statistical tools used to provide a better understanding of each strategy are the following. To assess the degrees of dependency between parameters and outputs, the partial rank correlation coefficients are calculated for each parameter–output response pair (Mckay et al., 1998). To determine whether the parameters can be optimized to produce the desired behavior, the evolutionary algorithm non-dominated sorting genetic algorithm II (NSGA-II) (Deb et al., 2002) has been used. More information for each tool can be found in Section 3.3.

In this paper, we have included figures that show key results from these analyses, which are then discussed in more detail. However, for completeness, we include the results of the emulation training and test procedures and all statistical analyses in the supporting website https://www.york.ac.uk/robot-lab/taskpartitioning/.

### 3.3 Statistical tests and techniques

This section shows the statistical tests used to analyze the results from the experiments. The statistical analysis is performed using Spartan (Alden et al., 2013); for more information about each technique, please refer to Alden et al. (2014) and Alden et al. (2018).

#### 3.3.1 Consistency analysis

**FIGURE 4:**
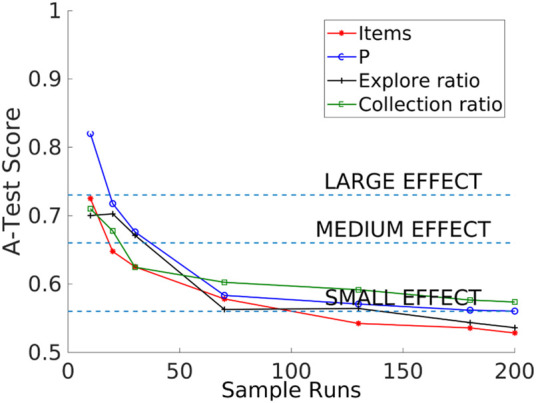
Consistency analysis that shows that the sample size value of 180 is large enough to avoid an aleatory uncertainty effect (A-test lower than 0.56) in the outputs (items collected, *P*, explore ratio, and collection ratio).

The consistency analysis technique allows the identification of the number of executions that minimizes the effect of aleatory uncertainty caused by inherent stochastic variation within non-deterministic simulations. In this technique, 20 distributions are compared with the Vargha–Delaney test for a different number of runs. The Vargha–Delaney A test is a non-parametric effect magnitude test that can be used to indicate the difference between two distributions (Delaney and Vargha, 2000). The more different the distributions are, the closer the score is to 0 and 1. We believe that our data do not need to be transformed as suggested in Neumann et al. (2015) because the amount of time that it takes for these variables to change is greater than the length of the tick used for simulations (0.1 s). The value of 180 as sample size has an A-test score below 0.56 as shown in Figure 4 which suggests that the aleatory uncertainty in the outputs has been mitigated and also avoids over-fitting the experiments with a larger sample size. In summary, this technique is used to minimize variation from non-determinism in the results to get the correct interpretation of the results.

#### 3.3.2 Partial rank correlation coefficients

The partial rank correlation coefficients (PRCCs) are used here in order to identify the degrees of dependency between each parameter. This is carried out by performing a Latin hypercube sampling across parameter space, and the number of samples is 1,000. The main difference between random sampling and Latin hypercube sampling is that with the latter, it is possible to increase the reliability that the entire space is covered adequately. The PRCC provides a measure of the influence of a single parameter with a single output. Strong correlations (close to 1 or −1) correspond to influential parameters over their respective outputs in spite of non-linearity introduced by other parameters. In summary, this technique allows us to identify the key parameters for specific outputs, and in this way, it is possible to identify if the error diversity or lack of it has any influence on these key parameters.

#### 3.3.3 Non-dominated sorting genetic algorithm II

The non-dominated sorting genetic algorithm II (NSGA-II) is an evolutionary technique that explores the entire parameter space in order to maximize and/or minimize multiple outputs. Once all the solutions for population converge (or meet the specific convergence criteria), this set of solutions is referred to as the Pareto front. In later sections of this paper, it will be revealed that the consideration of error diversity distorts this Pareto front, and this is correlated to the number of high-error robots.

### 3.4 Error model

**FIGURE 5:**
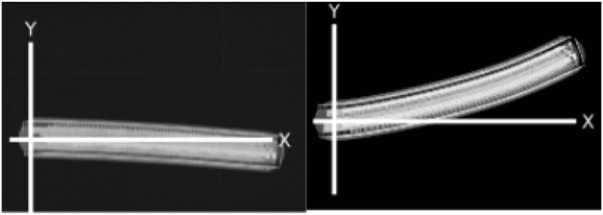
Trajectories of two different physical robots, A (left) and F (right), moving forward for 30 s with a speed of 0.02 m/s.

The error model consists of adding noise to each motor as shown in Eqs 3, 4. The simulated noise *μ* is generated from taking a sample from a Gaussian distribution each time tick with median *k*[*t*] and *σ* where *σ* changes for each robot. Curvature functions *k* for each robot can be found in the Appendix. Every time a robot changes the speed of its motors, timer *t* is set to 0. This noise model recreates the bias in the robot of moving toward a single direction. Examples of trajectories with physical robots are shown in Figure 5.

**FIGURE 6:**
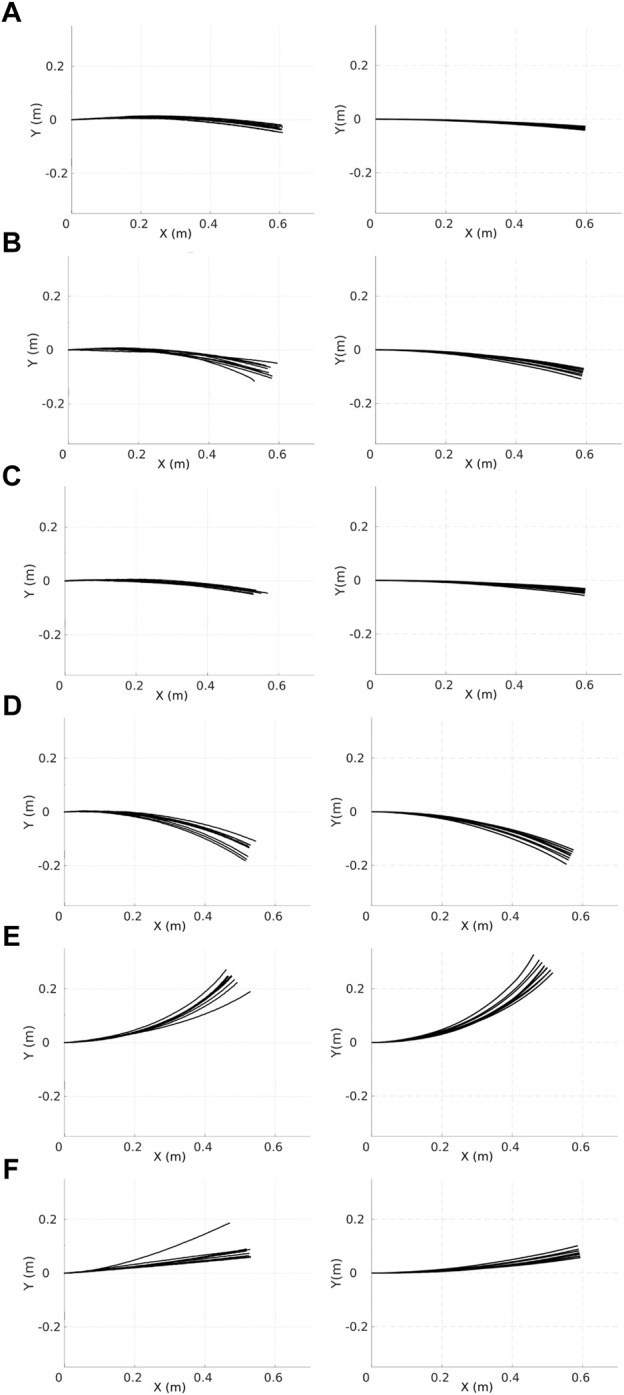
Trajectories of the physical (left column) robots and their respective simulated versions (right column). Each character (**A–F**) represents the ID of each different robot where, each robot moves forward along the *x*-axis for 30 s at a speed of 0.02 m/s, and 10 iterations are shown for each robot. X(m) and Y(m) represent the coordinates of the robot.

Experiments recorded with physical robots and trajectories produced in the simulator for the first six robots are shown in Figure 6. The error model in simulation closely resembles the error model in hardware. A model of the psi-swarm used in the simulator in this work can be found at https://github.com/edgarbuchanan/psiswarm_model.

$$\begin{array}{ll} rightWheelSpeed & =actuatedRightWheelSpeed\\ & \;\pm \mu *actuatedRightWheelSpeed, \end{array}$$ (3)

$$\begin{array}{ll} leftWheelSpeed & =actuatedLeftWheelSpeed\\ & \;\pm \mu *actuatedLeftWheelSpeed. \end{array}$$ (4)

It is important to mention that for the heterogeneous error, the error model for each robot is fixed, and it does not change for the experiment shown in this paper.

## 4 Experiments and results in a multi-scale model approach

This section presents experiments to demonstrate the performance difference between heterogeneous and homogeneous errors. First, by using emulation, a sensitivity analysis is performed for each strategy (NPS and DPS) from a macro perspective. Second, through experiments in simulations and hardware, the performance of the swarm from a micro perspective is considered.

### 4.1 A study from a macro perspective using emulation

This section presents the results from the emulation for each strategy (NPS and DPS).

#### 4.1.1 Non-partitioning strategy

The first strategy to be described is the non-partitioning strategy (NPS), where the robots take the items directly from their source and transport them to the home area. *P*_*i*_ is the same as *d*, and it does not change across the simulation.

For the NPS emulation, two parameters are considered: *swarm size* and *d*. The *swarm size* is the number of robots that comprise the swarm where the range is between 2 and 14 robots. Heterogeneous and homogeneous errors are considered for the emulation, in a range for *d* from 0.5 to 2.0 m. The width of the arena does not change, and the home area and items’ source are against the arena walls.

##### 4.1.1.1 Latin hypercube analysis

**FIGURE 7:**
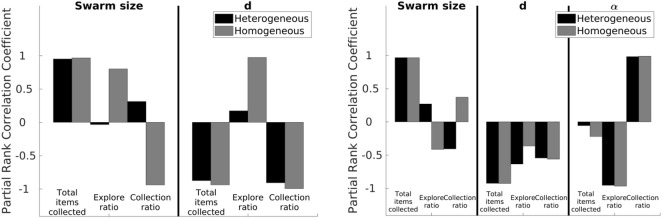
LHA for the NPS (left) and DPS (right) with homogeneous and heterogeneous error. Left: correlations change for each error type except for the *total items collected* for both parameters (*swarm size* and *d*) and the collection ratio for the *d* parameter. Right: results are similar between heterogeneous and homogeneous errors except for the correlation between the *swarm size* term, the *explore ratio*, and the *collection ratio*.

**FIGURE 8:**
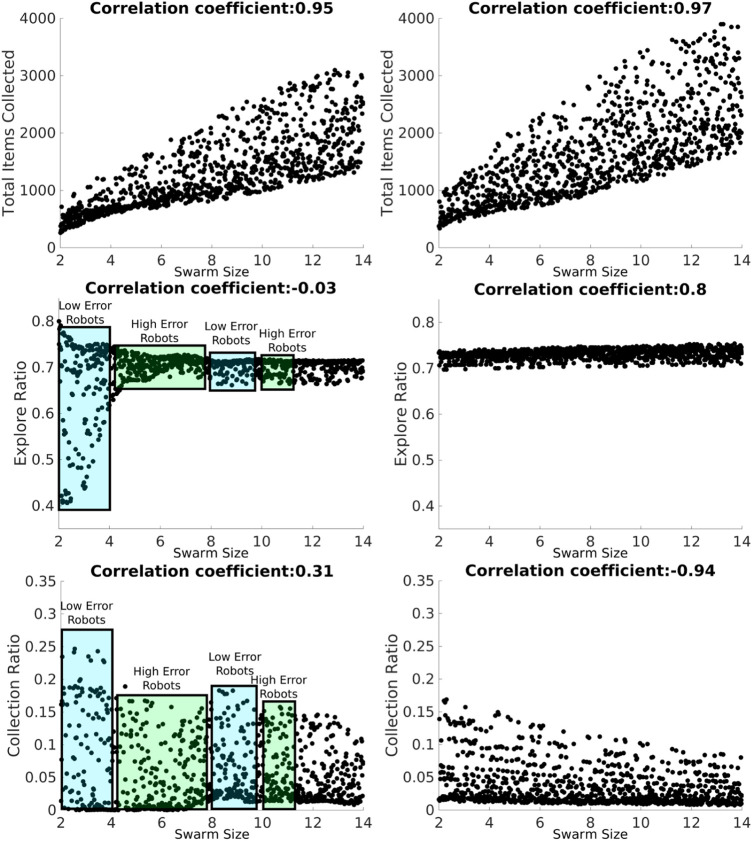
LHA for the *swarm size* term for the NPS with heterogeneous (left column) and homogeneous (right column) errors and for the *total items collected* (top row), *explore ratio* (middle row), and *collection ratio* (bottom row) outputs. The blue rectangles represent the proportion of low-error robots, and the green box represents the proportion of high-error robots. Robot individual error models introduce fluctuations with the heterogeneous error as shown with the *explore ratio* and *collection ratio*. However, this does not happen with the homogeneous error because all the robots share the same error parameters.

Results from the Latin hypercube analysis are illustrated in Figure 7 and summarized in Table 2, and key scatter plots are shown in Figure 8.

**TABLE 2:** LHA correlation values for the NPS. Values in bold represent a big difference, greater than 0.5, between heterogeneous and homogeneous errors.

Parameter—output	Heterogeneous	Homogeneous
*Swarm size—total items collected*	0.95	0.97
*Swarm size—explore ratio*	**−0.03**	**0.8**
*Swarm size—collection ratio*	**0.31**	**−0.94**
*d—total items collected*	−0.87	−0.94
*d—explore ratio*	**0.17**	**−0.99**
*d—collection ratio*	**−0.91**	**0.97**

Results for heterogeneous error show that the *swarm size* term is only highly positively correlated (absolute correlation coefficient greater than 0.7) with the *total items collected* output (Figure 7). This means that as the *swarm size* increases, the *total items collected* output increases, and this is because there are more robots collecting items in the arena.

The parameter *d* is highly negatively correlated with the *total items collected* and the *collection ratio*. As *d* decreases, it takes less time to transport the items from the items’ source to the home area. In addition, since the distance the robots are traveling is shorter, the probability of finding items increases.

Results for homogeneous error show that the *swarm size* term is not only highly correlated with the *total items collected* output but also with both the *explore ratio* and *collection ratio* (Figure 7, right). As the *swarm size* increases, time spent in the *explore* state increases because robots spend more time avoiding each other. As a consequence, robots travel more when transporting items. Therefore, the error increases and the probability of finding items decreases, which is reflected in the *collection ratio* output where it has a highly negative correlation with the *swarm size* term.

In a similar way, *d* is not only highly correlated with the *total items collected* and the *collection ratio* but also with the *explore ratio* for both error types. As *d* increases, the probability of finding items decreases; therefore, robots spend more time exploring than transporting items because they are getting lost more often.

Even though robots with homogeneous and heterogeneous errors are performing the same strategy, NPS, with the same settings, results from LHA differ. The main reason for this difference is that the individual errors in each robot, for the heterogeneous errors, affect the performance of the swarm as a whole in different ways, as explained below.

*Total items collected* are very similar for homogeneous and heterogeneous errors, as shown in Figure 8. However, as for the *explore ratio*, the correlation coefficient is very different from each other, −0.03 for heterogeneous error and 0.8 for homogeneous error. With the heterogeneous error model, there are fluctuations across the *swarm size* term space due to the individual errors considered. For example, between *swarm sizes* 2 and 3 (robots A, B, and C), the *explore ratio* ranges from 0.4 to 0.8. This is because this group of robots is characterized by their small errors compared to other robots in the swarm, as shown in the error models in the previous section (Figure 6). Therefore, these robots spend more time transporting items than exploring. However, this does not occur with the homogeneous error, as shown in Figure 8, where the *explore ratio* increases steadily without any oscillations present.

The *collection ratio* output for heterogeneous error also has fluctuations, as shown in Figure 8. In the regions where high-error robots are introduced, the *collection ratio* drops and increases again when low-error robots are introduced (i.e., the range between five and seven robots). However, with homogeneous error, the *collection ratio* decreases steadily with no fluctuations as the swarm density increases and the robots spend more time avoiding each other. This is due to there being no high-error robots that are introduced at any point because all the robots have the same error.

As shown, results are different from LHA between heterogeneous and homogeneous errors for the explore ratio and the collection ratios. This is because fluctuations introduced between high- and low-error robots affect the correlation coefficients. Complementary LHA scatter plots can be found online at https://www.york.ac.uk/robot-lab/taskpartitioning/.

##### 4.1.1.2 Non-dominated sorting genetic algorithm II

**FIGURE 9:**
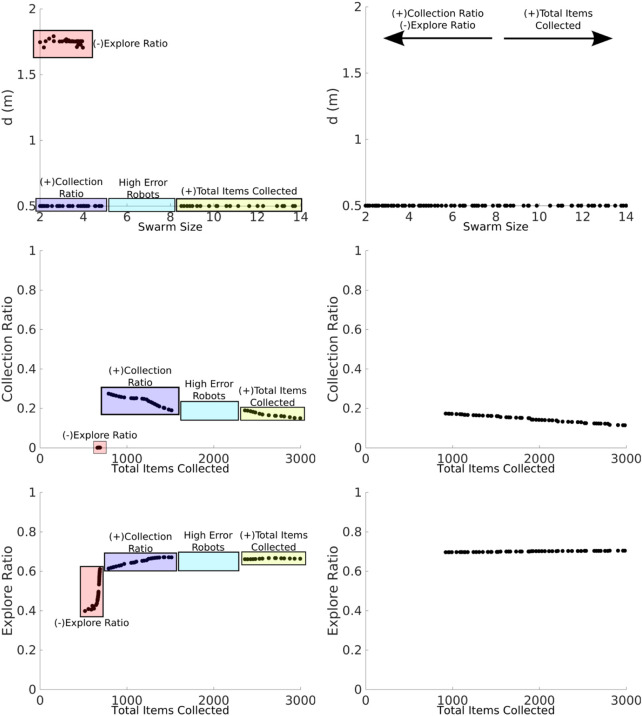
NSGA-II results for the NPS with heterogeneous (left column) and homogeneous (right column) errors. The two-dimensional inputs are *swarm size—d* (top row). Two-dimensional outputs are shown as *total items collected—collection ratio* (middle row) and *total items collected—explore ratio* (bottom row). With both the heterogeneous error and the homogeneous error, the *total items collected* are maximized with high *swarm sizes* (yellow box). With both the heterogeneous error and the homogeneous error, the *collection ratio* is maximized with low swarm sizes (blue rectangles). The *explore ratio* is maximized with low *swarm sizes* and high *d* for the heterogeneous error and low *swarm sizes* and low *d* for the homogeneous error. The latter result demonstrates that since this proportion of robots experiences a low error, it is better for these robots to travel a longer *d*, and as the robot density is lower, the robots spend less time avoiding each other. The Pareto front is discontinuous for the heterogeneous error which means that these robots with high-error rates harm the item collection than they contribute to it.

The NSGA-II was used to find the Pareto front for the best values for *swarm size* and *d* parameters in order to maximize the *total items collected* and the *collection ratio* and minimize the *explore ratio*, as shown in Figure 9. The Pareto front is discontinuous for the heterogeneous error (left column), as the emulation captures the heterogeneity in the errors. For instance, for small swarm sizes, from 2 to 3 (robots A, B, and C), the error is small, which means that the robots can travel longer distances. This minimizes the *explore ratio*, and they spend the least amount of time avoiding each other since the swarm density is low. Robots between *swarm sizes* 2 and 4 with a *d* of 0.5 maximize the collection ratio. This is because the robots are traveling a shorter distance between home and source; therefore, error accumulation is small. Between 5 and 7 (robots E, F, and G), robots are characterized by their high degree of error. Therefore, these robots behave more like obstacles and impede item collection. As a consequence, the swarm is affected negatively. Lastly, between 8 and 14 (robots H, I, J, K, L, M, and N), robots with low error are again introduced, which contribute to a high *total items collected* output. Further work could potentially use this discontinuous Pareto front in order to identify the threshold of the number of high-error robots that when overcome the swarm throughput is affected negatively. This would help to measure the degrees of robustness of the task.

The Pareto front is continuous for the homogeneous error (right column), as all the robots contribute in the same way. If the *total items collected* were to be maximized, the *swarm size* needs to be increased. However, if the *collection ratio* is maximized and the *explore ratio* is minimized, the size of the *swarm* needs to be decreased. In order to optimize the three outputs, it is necessary to have the smallest *d*.

##### 4.1.1.3 Summary

From this analysis, it can be concluded that it is important to consider heterogeneous and homogeneous errors, as the results can be misleading if they are considered separately because of the different correlation values, and this validates the first hypothesis. In addition, the use of different statistical techniques helps provide a better, more in-depth understanding of the system. The NSGA-II exploits the heterogeneity and finds new solutions that maximize and minimize outputs and strategies described in the following section to decrease this effect by dividing the task into multiple components.

#### 4.1.2 Dynamic partitioning strategy

The dynamic partitioning strategy (DPS) is a strategy where robots change their individual partition length (*P*_*i*_) according to a penalty and reward mechanism. *P*_*i*_ changes with the parameter *α*.

*α* regulates the amount of penalty and reward to *P*_*i*_. As *α* increases, the robot *i* gets penalized and not rewarded, and as *α* decreases, the robot is rewarded more than penalized. The interval used for the experiments shown in this section is [0, 1]. Initial *P*_*i*_ is randomly selected from a uniform distribution at the same interval as *d*.

##### 4.1.2.1 Latin hypercube analysis

**FIGURE 10:**
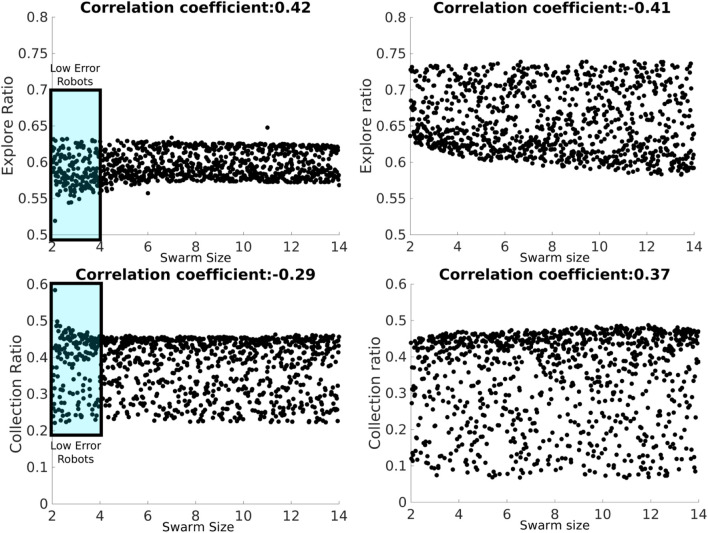
LHA for the *d* parameter for the DPS with heterogeneous (left column) and homogeneous (right column) errors and the explore ratio (top row) and collection ratio (bottom row) outputs. The blue rectangle represents the proportion of low-error robots.

Results from the Latin hypercube analysis are summarized in Table 3, and key scatter plots are shown in Figure 10.

**TABLE 3:** LHA correlation values for the DPS. Values in bold represent a big difference, greater than 0.5, between heterogeneous and homogeneous errors.

Parameter—output	Heterogeneous	Homogeneous
*Swarm size—total items collected*	0.9	0.97
*Swarm size—explore ratio*	**0.42**	**−0.41**
*Swarm size—collection ratio*	**−0.29**	**0.37**
*d—total items collected*	−0.76	−0.93
*d—explore ratio*	−0.73	−0.36
*d—collection ratio*	−0.42	−0.56
*α* *—total items collected*	0.07	−0.22
*α* *—explore ratio*	−0.97	−0.97
*α* *—collection ratio*	0.99	0.99

Results between heterogeneous and homogeneous errors are very similar for all the correlations, except for the correlation between the *swarm size* term and the *explore ratio* and the *collection ratio*, as shown in Figure 7. This discrepancy is produced by the heterogeneity within the individual errors. However, the correlation is kept low with the absolute correlation coefficient being lower than 0.7.

The *total items collected* output is, once again, mainly correlated to the *swarm size* and *d* parameters for the reasons explained in the previous section. However, *α* has a negative correlation with the *explore ratio* and a positive correlation with the *collection ratio*. This means that as *α* increases, the robots are exploring less and finding items more often because all the robots are adjusting their *P*_*i*_. In other words, as *α* increases, robots are traveling shorter distances, allowing for less dead-reckoning error accumulation.

The discrepancy mentioned earlier for the *swarm size* term is shown in Figure 10. For the heterogeneous error, the *collection ratio* decreases and the *explore ratio* increases, whereas for the rest of the swarm, this correlation pattern cannot be seen. This could be due to high error robots compensating for the heterogeneous error, whereas for the homogeneous error, the *explore ratio* and the *collection* ratio decrease and increase steadily, respectively, for the homogeneous error. As the swarm size increases, there is a higher supply of robots, which allows for the successful creation of a chain between the home area and the source.

Fluctuations introduced by low-error robots affect the estimation of the correlation coefficient.

##### 4.1.2.2 Non-dominated sorting genetic algorithm II

**FIGURE 11:**
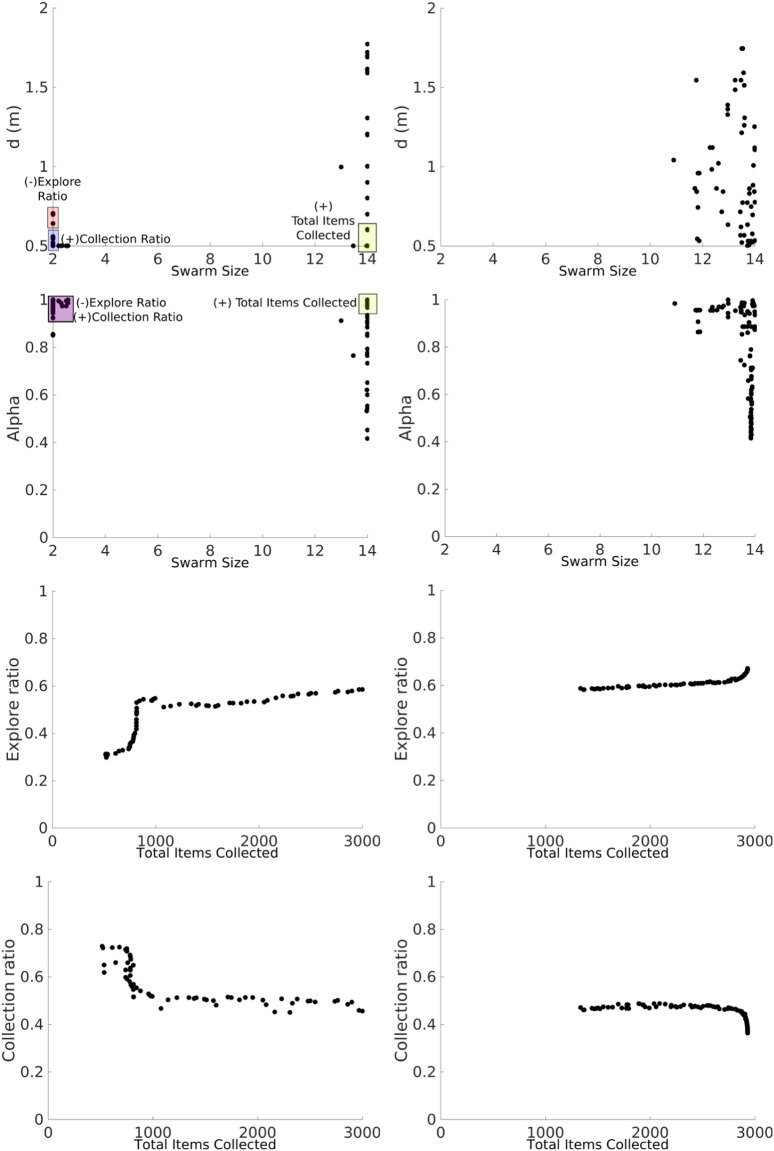
NSGA-II results for the DPS with heterogeneous (left column) and homogeneous (right column) errors. Two-dimensional inputs are shown as *swarm size—d* (first row) and *swarm size—alpha* (second row). Two-dimensional outputs are shown as *total items collected—explore ratio* (third row) and *total items collected—collection* ratio (fourth row).

Large swarm sizes provide the best item collection, and *α* regulates the *explore ratio* and *collection ratio* where as *α* increases, the *collection ratio* increases and the *explore ratio* decreases; see (Figure 11) for this effect with the heterogeneous error. In a similar way, the NSGA-II for the heterogeneous error exploits the small swarm size in order to achieve the best *explore ratio* and *collection ratio*.

The Pareto front for the DPS is smooth, and discontinuities, or gaps, are smaller than those in the NPS. This is because the DPS has better regulation over individual errors, homogenizing the swarm. Each robot *i* is learning its own *P*_*i*_ for the given *alpha* (see next section for more details). This can also be seen for the *α* and *swarm size* inputs where there are only two batches of solutions: the ones closer to the swarm size of two robots and the ones closer to the swarm size of 14. This means that fluctuations by high- and low-error robots between the intervals 4 and 10 are ignored and have no significant contributions to the results.

Results for the homogeneous error show that a larger swarm provides the best item collection, and as *α* increases, the *collection ratio* increases, while the *explore ratio* decreases. The Pareto front for the set of solutions is continuous and smooth.

##### 4.1.2.3 Summary

*α* helps to regulate the *collection ratio* in order to optimize the *explore ratio*. The *total items collected* output is provided mainly by the *swarm size* and *d*. In this way, it is possible to find a set of parameters that provides a good trade-off between the *explore ratio* and the throughput of items according to the needs of the user. Finally, fluctuations caused by individual error models are reduced, and a continuous Pareto front for the output is generated for the heterogeneous error due to individual convergence of *P*_*i*_ for each robot. Since the robots are traveling shorter distances, the error is lower and more uniform. More information about *P*_*i*_ convergence can be found in the experiments from simulation and hardware in the next section.

### 4.2 A study from a micro perspective using a simulator and hardware

In the previous section, the effect of considering individual robot errors in emulation with each strategy was explored. An emulator was used to aid with this study, and it was found that results differ for heterogeneous and homogeneous errors. In this section, this discrepancy is explored in detail by using experiments from a micro perspective with simulations and hardware (stages 2 and 3 from the experimental framework shown in Figure 2).

The parameter values chosen, unless stated, for the experiments in this section are shown in Table 4.

**TABLE 4:** Parameter values for micro perspective experiments.

Parameter	Value
Experiment length	5 h
Swarm size	6
*d*	1 m
*P*	0.5 m
*α*	0.5

#### 4.2.1 Heterogeneous and homogeneous errors

**FIGURE 12:**
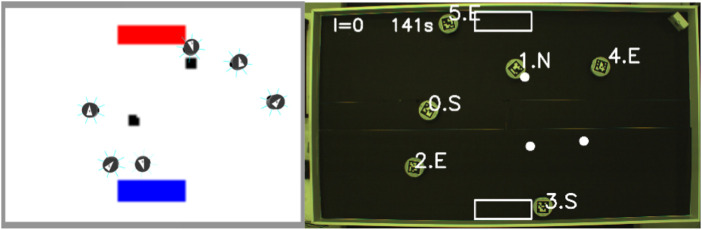
Screenshot of one of the experiments in simulation (left) and hardware (right).

As discussed in Section 3.4, when modeling the error for each robot, it was found that the error varies between robots. This section describes error diversity and non-diversity in simulations (Figure 12).

**FIGURE 13:**
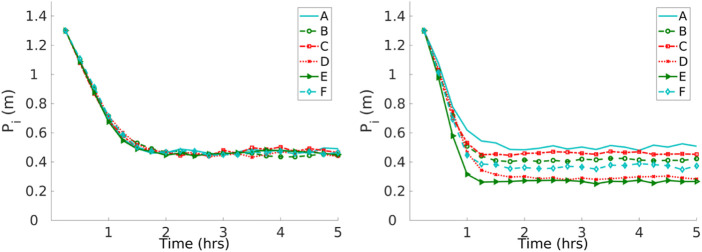
Convergence to a solution with homogeneous (left) and heterogeneous (right) errors with the DPS. Robots with the homogeneous error converge to a single *P*_*i*_ and robots with the heterogeneous error converge to different *P*_*i*_.

In the first set of experiments, the robots are performing DPS, where *P*_*i*_ is the same as *d*. The convergence of *P*_*i*_ is shown in Figure 13. All the robots converge to a single similar *P* with the homogeneous error. However, robots with heterogeneous errors converge to a different *P*_*i*_ for each robot *i*.

**FIGURE 14:**
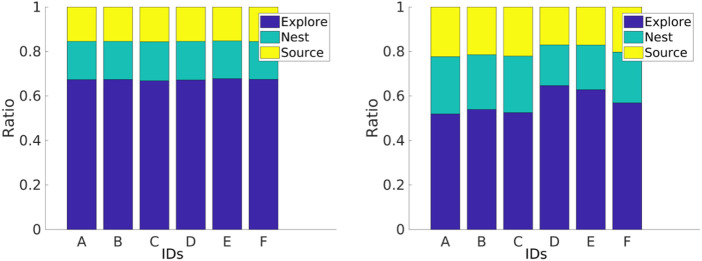
Individual performance with homogeneous (left) and heterogeneous (right) error models. Robots with the homogeneous error have similar individual performance, and robots with the heterogeneous error have different individual performance.

The impact of different errors is reflected in individual performance. In experiments shown in Figure 14, all robots start with a *P*_*i*_ of 0.4 m and *α* of 0.5 with homogeneous and heterogeneous error models for DPS. The robots with homogeneous errors have a similar individual performance to each other. However, robots with heterogeneous errors have different individual performance according to each robot.

Robot specialization emerges from DPS in the sense that every robot learns its own *P*_*i*_ according to its inherent degree of error, as shown in Figure 13. This is consistent with the results presented in Figure 14, where the *P*_*i*_ for each robot is correlated with the amount of time that a robot spends exploring the environment. For example, robot A spends the least amount of time in the explore state and, therefore, has the greater *P*_*i*_ with a value close to 0.5 m. Robots D and E spend the greatest amount of time in the explore state, and these robots travel the small *P*_*i*_ with a value close to 0.3 m. In addition, this is consistent with the trajectories shown in Figure 6.

For instance, Figure 13 shows that robots D, E, and F learn the smallest *P*_*i*_, and this is related to their individual performance, where these robots spend more time in the *explore* state than the rest of the robots, as shown in Figure 14. In addition, Figure 6 shows that these same robots have bigger error drifts.

**FIGURE 15:**
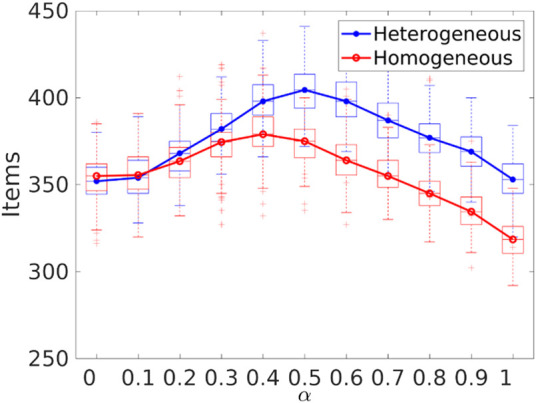
Item collection with homogeneous and heterogeneous errors with different values for *α*. The peak for item collection is 0.4 for the homogeneous error model and 0.5 for the heterogeneous error model.

It is important to bear in mind the difference in performance between heterogeneous and homogeneous errors in order to select the appropriate value of *α* for the swarm to maximize the item collection, as shown in Figure 15. In the case of a homogeneous error model, *α* should be 0.4 in order to have the best item collection. As with the heterogeneous error model, the value would be 0.5.

**FIGURE 16:**
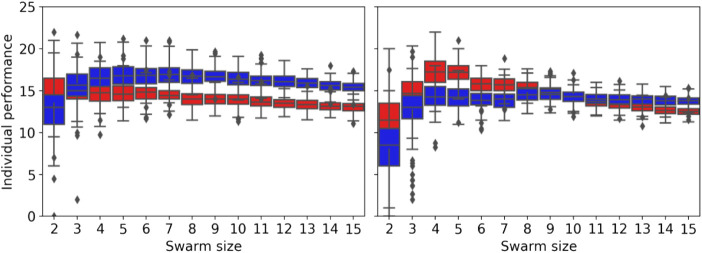
Individual performance for different swarm sizes for the homogeneous error (left) and heterogeneous error (right) for the NPS in red and the DPS in blue. The individual performance decreases after the first robot for the NPS and after seven robots for the DPS with the homogeneous error. The individual performance peaks at four robots for the NPS and at eight robots with the DPS for the heterogeneous error.

Figure 16 shows the individual performance for different swarm sizes, strategies (NPS and DPS), and error types (homogeneous and heterogeneous). The individual performance is estimated by dividing the *total items collected* by the *swarm size*. For the *homogeneous error*, the individual performance starts to drop after swarm size 2 for the NPS and after a swarm size of 7 for the DPS. The results differ with the *homogeneous error*, and this is due to the mix of low- and high-error robots in the swarm, as mentioned in the previous section. For example, NPS peaks at four robots because the first four robots experience low error, and after this, the robots introduced experience high error, as shown in Figure 8. This is not the case for the DPS, where the distribution resembles more of a bimodal distribution with two peaks at 4 and 9, each peak at the location where robots with low error are introduced.

More results regarding the robot swarm flexibility against a changing *d*, change of error models, and change of *swarm size* can be found in the supplementary material https://www.york.ac.uk/robot-lab/taskpartitioning/.

Indirect item transference is the method chosen for most of the experiments shown in this section because it has the highest item collection with the lowest convergence speed. This is an important aspect to consider due to the battery life of the physical robot only lasting 30 min.

To conclude, the aforementioned results demonstrate that each robot exhibits different performance, and this validates the second hypothesis. It is important to consider individual models for each robot when working with swarm robotics. By doing so, it is possible to reduce the reality gap and select the best *α* that maximizes the item collection, as well as potentially select the best *P*_*i*_ and *α* for each robot to improve the item collection. In Section 4.2.3, experiments with physical robots and how they compare with simulations are shown.

#### 4.2.2 Social entropy

Results from the NSGA-II in emulation for the NPS showed a discontinuous Pareto front for the heterogeneous error and a continuous Pareto front for the homogeneous error (Figure 9). In this section, these results are explored in more detail to understand the reason for this discrepancy, using an approach based on a social entropy metric. This metric measures the error diversity across the swarm and is described in Section 3.1.

**FIGURE 17:**
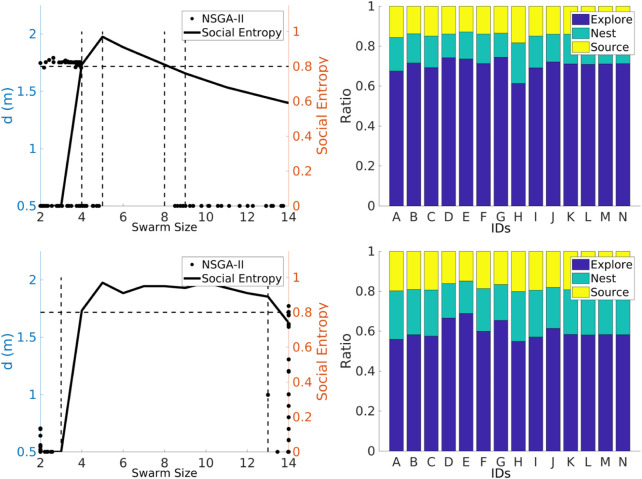
Social entropy and individual performance (left and right columns) for each strategy: NPS and DPS (first and second rows, respectively). Social entropy changes for each strategy due to the number of robots classified as having high error.

Decision trees and nearest neighbor classifiers are used to categorize robots into two groups according to their individual performances. Principal component analysis (Wold et al., 1987) is used to convert the variables from the individual performance data to their principal components. This is carried out in order to transform possibly correlated variables into a set of values for linearly uncorrelated variables. The requirement for classification is that the accuracy should be higher than 90%. Figure 17 illustrates the social entropy for each swarm size in the interval [2, 14] for each strategy. The social entropy is overlapped with the results from the NSGA-II reported in Section 4.1.

The social entropy for the NPS illustrates that the Pareto front breaks when the social entropy reaches its highest peak. For a swarm interval between 2 and 4, the social entropy is low. This means that the swarm is homogeneous and composed of robots with a low degree of error. This is the reason for the cluster of points in *d* at 1.75 m. However, after the first robot with a high degree of error is introduced (robot D or the fourth robot), the social entropy increases. At this stage, the swarm is homogeneous enough to have a continuous Pareto front. However, the discontinuity in the Pareto front appears when the second robot with a high degree of error is introduced (robot E or the fifth robot). The social entropy reaches its highest peak point at this point. The social entropy starts to decrease after the sixth robot (robot F) is introduced. The social entropy decreases steadily because more robots with a low degree of error are added to the swarm, which decreases diversity. The Pareto front reappears after the ninth robot is introduced. After this point, the Pareto front is similar to the homogeneous error Pareto front (Figure 9).

Overall, the social entropy across the swarm for the DPS is similar to the NPS. The social entropy peak again is located after the fifth robot, and the Pareto front becomes discontinuous after this point. However, in contrast to the NPS, the social entropy remains high (greater than 0.8). This prevents the Pareto front from being continuous until the social entropy drops below 0.8. The DPS creates a clear distinction between high and low degree of error, and because of this, the social entropy increases. This might be because task partitioning is sensible for high noisy degree of error robots.

As noted, the social entropy changes with each strategy, and the reason is that each strategy provides a different performance for each robot. For the NPS, the highest degree of error robots classified are 2, robots D and G. For the DPS, the robots experience the least amount of time in the explore state. The number of high-degree error robots increases to 4 (D, E, G, and J).

In the case of the DPS, robots D, E, G, and J experience the highest degree of error. Therefore, these robots cannot be further optimized. This is consistent with the results shown in Figure 17, where the *P*_*i*_ for robots D and E with a *d* of 0.4 m does not increase indefinitely as with robots A, B, C, and F. This means that robots have not reached the optimal distance that decreases the error. In other words, robots D, E, G, and J experience such a high degree of error that the partitioning strategies are unable to provide a suitable *P*_*i*_ for the robots to increase their performance.

#### 4.2.3 Experiments with physical robots

In this section, the experiments studied in the previous section are explored with physical robots. Three different arena sizes are used for the experiments using six robots, where individual performance and convergence are explored. A single replicate for each arena is shown in this section. It is important to mention that a definite conclusion cannot be drawn from the results shown in this section due to this low sample size; however, the results are promising. The results shown are not the same. A screenshot from an experiment is shown in Figure 12.

**FIGURE 18:**
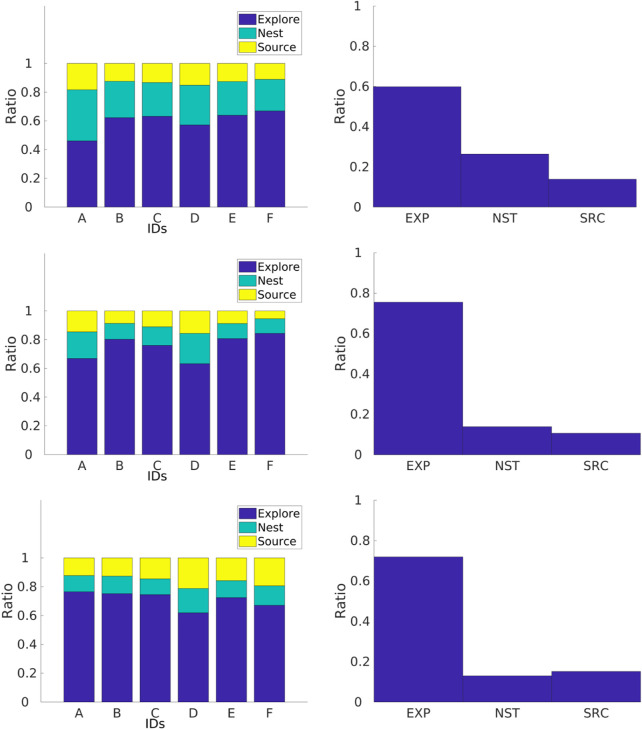
Individual performance for physical robots performing the NPS in three different arenas with dimensions of 1.0 m × 1.3 m (top row), 2.1 m × 1.2 m (middle row), and 2.4 m × 1.6 m (bottom two). The go-to-source rate is smaller than the go-to-nest rate as the arena becomes smaller.

Results for robots performing the NPS are shown in Figure 18. Regardless of the arena size, the robots experience different individual performance which means that they are not identical to each other. In addition, robots spend most of their time exploring instead of retrieving items in the home area. Finally, robots spend less time in the *go-to-source* state than in the *go-to-nest* state. This is because since robots are traveling a longer *P*_*i*_, they experience a greater drift from the original position, causing the target to be beyond the walls of the small arena, as previously seen. This effect decreases as the arena size increases because the target is within the arena boundaries.

**FIGURE 19:**
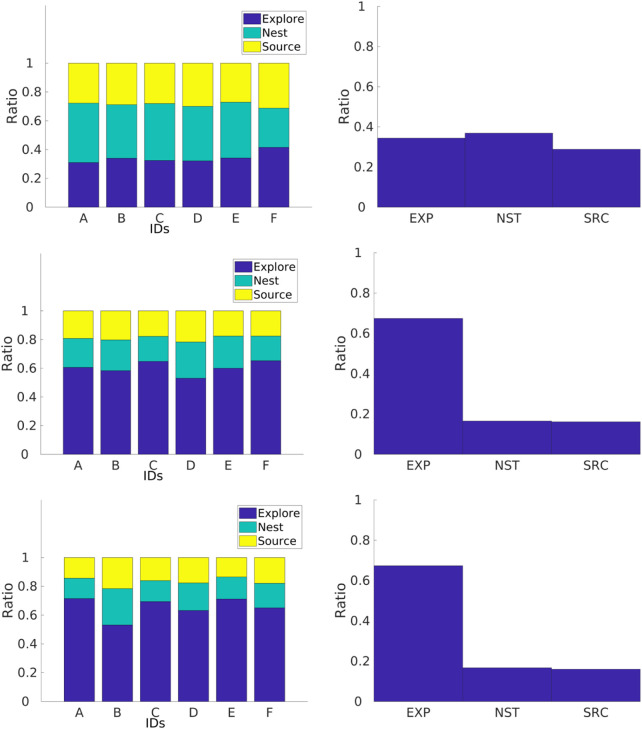
Individual performance for physical robots performing the DPS for three different arenas with dimensions of 1.0 m × 1.3 m (a), 2.1 m × 1.2 m (c), and 2.4 m × 1.6 m (e). Time spent in the *go-to-source* state is roughly similar to that in the *go-to-nest* state, and time spent in the *explore* state is lower than that with the DPS.

Individual performance for robots with the DPS can be found in Figure 19. In a similar way to experiments with the NPS, robots performing the DPS experience an increment in the amount of time spent in the *explore* state as the arena size increases. In contrast with the NPS, robots spend more time in the *going-to-source* state. The reason for this is that the robots are traveling shorter *P*_*i*_ which decreases the probability of the target being beyond the arena size for the small arena in a similar way to that in the simulations.

**FIGURE 20:**
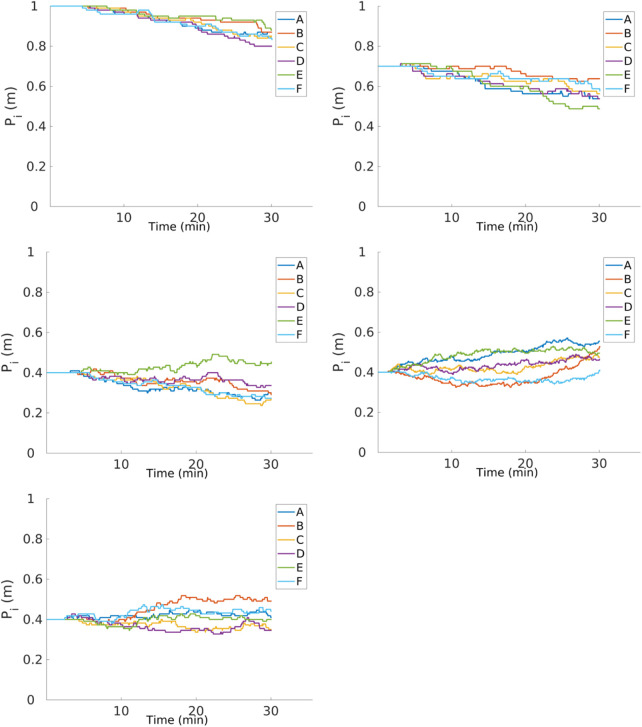
Convergence of *P*_*i*_ for different $P_{i}^{0}$ of 1.0 (top left), 0.7 (top right), and 0.4 m (middle left) for an arena of 2.1 m × 1.1 m. Convergence for arenas 1.0 m × 1.3 m (middle right) and 2.4 m × 1.6 m (bottom left) for $P_{i}^{0}$ of 0.4 are also shown. The convergence changes from robot to robot and is due to the error and velocity of the robot.

Since the battery of these robots last for 30 min, the convergence experiments were divided into three different sets as shown in Figure 20. In each set, the robots started with a different *P* of 1, 0.7, and 0.4 m for an arena of size 2.1 m × 1.2 m. All the robots converge at a distance close of about 0.4 m, which varies from robot to robot. The velocity of convergence is not only related to the degree of the error in the robot but also to the speed of the robot which changes slightly for each robot. Furthermore, from the figures, it can be seen that there is no change in the *P*_*i*_ for at least for the first 5 min of the experiments. This is the time that it takes to find the first item.

**FIGURE 21:**
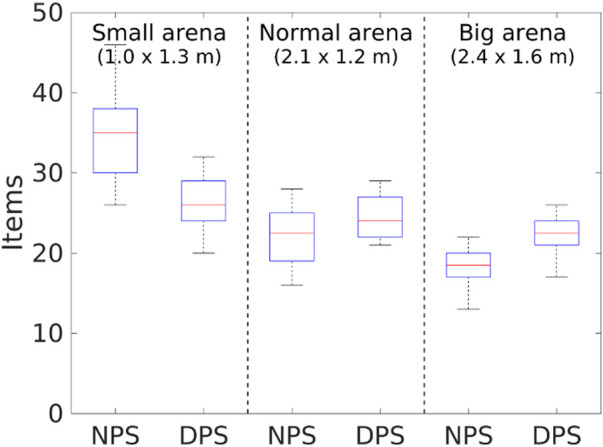
Item collection with physical robots and different arena sizes. The DPS starts to overcome the NPS as the arena size increases.

As for the item collection, the DPS performs better than the NPS as the arena size increases as shown in Figure 21 because of the following issues. First, as the area of the arena decreases, the probability of finding the items’ source increases even though the collection rate is low. This means that partitioning is not required. Second, robots are spending more time dropping and picking up items with the DPS, which causes a delay in the item getting to the nest. Finally, robots spend more time avoiding each other when they are in the *go-to-nest* state.

In summary, results from experiments with physical robots are similar to the results from simulation. The shape of the arena affects the performance of the *go-to-source* state, mainly for the NPS. The DPS performs better than the NPS as the size of the arena increases. Finally, the final *P*_*i*_ changes according to the amount of error in the robot *i*. However, there are some differences due to the reality gap because the models used in the simulator do not provide enough information about the real world.

## 5 Discussion

It is a common assumption that all the robots are similar, and a single robot model in simulations represents the entire group of robots in a robotic swarm. Furthermore, it was assumed that all robots would have the same behavior when performing a task. However, the work in this paper has shown that this is not necessarily the case.

At the moment of retrieving the trajectories for each robot, as shown in Section 3.4, each robot experiences a different degree of error. Each robot has a different bias, small or large, for moving in the left or right direction. The trajectories of 14 robots were modeled and recorded.

In the enriched analysis in Section 4.1, it was shown that two different patterns emerged from heterogeneous and homogeneous errors for each strategy. Each pattern represents different properties of the task partitioning approach. On one hand, the pattern shown with the homogeneous error provides information about the interactions of each parameter with each output. On the other hand, the pattern with the heterogeneous error provides information on how the swarm copes with robots with a high and low degree of error for each strategy.

The individual contribution of each robot changes according to its inherent error, as shown in Section 4.2. If the homogeneous error is considered, all the robots converge to the same *P*_*i*_ value. However, if the heterogeneous error is considered, then each robot converges to a different *P*_*i*_. *P*_*i*_ is able to adapt to changes in the error itself, *d*, and swarm size.

The social entropy measurement for each strategy bridges the results between the macro and micro perspectives (Section 4.2.2). The number of high-degree error robots plays an important role in defining optimality. As the number of high degrees of error robots increases, this affects the range of optimal solutions. In addition, even though the task partitioning strategies increase the performance of each robot, there are a specific number of robots that gain no benefit from task partitioning due to their high degree of error.

Finally, the experiments with physical robots shown in Section 4.2.3 validate the previous results in terms of the fact that each robot performs differently and each robot converges to a different solution according to the inherent error.

The work presented in this paper is limited to a single task, a single robot platform, and a single type of error, but there is no reason this work could be applied to other domains. Here are some examples of future work:

• The work presented in this paper could be extended to other tasks such as collective object transportation and collective decision-making. In collective object transportation, there is a high reliance on the input from the sensors, and the error in the sensors could be studied. In collective decision-making, error diversity could lead to false positives in decisions.
• The error diversity could be analyzed for other robot platforms, and this could lead to interesting results. On the one hand, the error diversity is so uniform that the approach in this paper becomes insignificant; on the other hand, the error diversity could be greater, and its importance becomes greater.
• In this paper, it was shown that the number of high-error robots changes the dynamics of the swarm’s behavior. Swarm diagnosis and recovery can be implemented to address these robots’ errors.

In conclusion, it is important to consider both homogeneous and heterogeneous errors in order to have a comprehensive understanding of the performance of a swarm.

## 6 Conclusion

The task partitioning strategies have been shown to increase performance in foraging tasks in robotic swarms with dead-reckoning noise (Pini et al., 2013; Pini et al., 2014; Buchanan et al., 2016). However, a common assumption is that all the robots in a swarm share the same error model. In this paper, how different degrees of error affect the swarm was studied.

In this paper, it has been shown how each robot in the swarm experiences different degrees of error (heterogeneous error). There is a single degree of error that describes all the robots (homogeneous error), and the results undertaking a foraging task differ when considering heterogeneous and homogeneous errors. The work has shown that it is important to consider both error types to have a full understanding of the system. Finally, the number of high degrees of error in the swarm defines optimality in the system. The degrees of robustness can be measured by identifying the ranges of optimality, and the reality gap is able to be reduced. The work in this study has also shown that it is possible to distinguish robots that harm the performance of the swarm.

Further work will consider measuring the degrees of robustness in tasks other than foraging. It will be important to examine how a range of different degrees of error would affect fault detection and diagnosis. Our hypothesis is that the number of false positives would increase and a threshold that differentiates robots between faulty and non-faulty would be required.

## Data Availability

The datasets presented in this study can be found in online repositories. The names of the repository/repositories and accession number(s) can be found in the article/Supplementary Material.
